# I am having trouble keeping up with virtual teaching activities: Reflections in the COVID-19 era

**DOI:** 10.6061/clinics/2020/e1945

**Published:** 2020-04-30

**Authors:** Renato Assis Machado, Paulo Rogério Ferreti Bonan, Danyel Elias da Cruz Perez, Daniella Reis Barbosa Martelli, Hercílio Martelli-Júnior

**Affiliations:** I Departamento de Diagnostico Oral, Faculdade de Odontologia, Universidade de Campinas (FOP-UNICAMP), Piracicaba, SP, BR; II Hospital de Reabilitacao de Anomalias Craniofaciais, Universidade de Sao Paulo, Bauru, SP, BR; III Centro de Ciencias da Saude, Universidade Federal da Paraiba, Joao Pessoa, PB, BR; IV Departamento de Clinica e Odontologia Preventiva, Faculdade de Odontologia, Universidade Federal de Pernambuco, Recife, PE, BR; V Diagnostico Oral, Curso de Odontologia, Universidade Estadual de Montes Claros (UNIMONTES), Montes Claros, MG, BR; VI Centro de Anomalias Craniofaciais, Curso de Odontologia, Universidade de Alfenas, Alfenas, MG, BR

To the Editor,

In this period of the COVID-19 pandemic, thousands of health schools have bolted into action to maintain theoretical learning activities. With the help of information technology and pedagogical sectors, online manuals and guides are being created so that professors and students have access to them in real-time. The field of medicine has not been any different. Electronic platforms, video conferencing rooms, and social media are being used for daily teaching purposes. Electronic educational platforms are the official mediums that educational institutions present as an alternative to face-to-face activities 1.

In theory, we have been able to adapt these practices for teaching medicine. However, the title of this text (“*I am having trouble keeping up with virtual teaching activities”*) stems from a recurring complaint, and it elicits a deep reflection on whether we, professors and students, are capable of assimilating extensive knowledge in a short period while remodeling the entire education system.

We know that medical programs involve several theoretical, laboratory, and clinical training components. This training is traditionally conducted in person, especially laboratory and clinical activities. The support and follow-up provided by teachers to students for theoretical and practical aspects of laboratory and clinical activities are fundamental for successful learning. However, how can one hold the student's attention with only virtual theoretical classes? A technique widely used for self-directed learning is the inverted classroom model (ICM). In this blended-learning method, the self-directed learning phase (individual phase) precedes the classroom-instruction phase. Furthermore, in one of the ICMs being used in these circumstances, learning takes place in small groups in problem-based-learning scenarios. However, generally, these methodologies were first used virtually and subsequently applied in the classroom, and with a small group 2. Transferring these methodologies to a larger group of students requires more professors, computer technicians, and equipment. The financial positions of medical schools vary considerably worldwide. Particularly for digital medical teaching, the whole slide imaging systems are relatively expensive, in addition to the need for a good hardware structure. Moreover, in many schools, these changes were not proposed at all.

As part of social distancing measures, professors have had to relocate their classes and other activities, including research practices and clinical care, to online platforms in a short time, but not all of it is feasible. In many countries, such teaching through virtual platforms has raised several difficulties, including the quality and speed of internet services and overload on some platforms in use. This unique moment in the history of humanity provokes different reflections. Analyzing the projections of the transmission dynamics of the novel coronavirus in the absence of effective preventive measures, a recent study predicts the need for prolonged or intermittent isolation and social distancing measures until 2022 3. We are aware of the expertise of medicine professors. Currently, in order to maintain the quality of online medical education, efforts must be devoted to investing in appropriate equipment and personnel.

## References

[1] Rose S (2020). Medical Student Education in the Time of COVID-19. JAMA.

[2] Tolks D, Schäfer C, Raupach T, Kruse L, Sarikas A, Gerhardt-Szép S (2016). An Introduction to the Inverted/Flipped Classroom Model in Education and Advanced Training in Medicine and in the Healthcare Professions. GMS J Med Educ.

[3] Kissler SM, Tedijanto C, Goldstein E, Grad YH, Lipsitch M (2020). Projecting the transmission dynamics of SARS-CoV-2 through the postpandemic period. Science.

